# Swinging Mass Through the Pulmonary Valve: A Rare Case of Right Ventricular Myxoma

**DOI:** 10.3390/life15111750

**Published:** 2025-11-14

**Authors:** Cristiana Bustea, Andrei-Flavius Radu, Paula Bianca Maghiar, Roxana Brata, Elena Emilia Babes

**Affiliations:** 1 Doctoral School of Biomedical Sciences, University of Oradea, 410087 Oradea, Romania; cbustea@uoradea.ro (C.B.); brata.roxanadaniela@didactic.uoradea.ro (R.B.); eebabes@uoradea.ro (E.E.B.); 2 Department of Preclinical Disciplines, Faculty of Medicine and Pharmacy, University of Oradea, 410073 Oradea, Romania; 3 Department of Psycho-Neurosciences and Recovery, Faculty of Medicine and Pharmacy, University of Oradea, 410073 Oradea, Romania; 4 Department of Surgical Disciplines, Faculty of Medicine and Pharmacy, University of Oradea, 410073 Oradea, Romania; 5 Department of Medical Disciplines, Faculty of Medicine and Pharmacy, University of Oradea, 410073 Oradea, Romania

**Keywords:** cardiac myxoma, right ventricle, pulmonary embolism, surgical intervention

## Abstract

Primary cardiac tumors are rare, with an estimated incidence of 0.001% to 0.3% in autopsy series. Most are benign, the most common being cardiac myxomas, which typically originate in the left atrium. Right ventricular myxoma is among the rarest primary cardiac tumors, and its true incidence is difficult to determine, as most data come from isolated case reports. This paper aims to report a case of right ventricular myxoma in a young woman with a history of childhood malignancy and to discuss the possible association between the two conditions. Echocardiography, thoracic computed tomography (CT), and pulmonary CT angiography were used to assess the presence, location, and size of the tumor. The definitive diagnosis was established by histopathological examination. A 34-year-old woman, with a past medical history of acute lymphoblastic leukemia (ALL) in childhood, presented with a dry cough and exertional dyspnea persisting for three weeks. Transthoracic echocardiography revealed a mass located in the right ventricular outflow tract (RVOT), attached near the tricuspid valve and intermittently prolapsing into the pulmonary trunk. CT imaging confirmed the presence of the tumor in the RVOT and the main pulmonary artery. Because of the high risk of massive pulmonary embolism, the patient underwent urgent surgical excision of the tumor. Histopathological analysis confirmed the diagnosis of cardiac myxoma. The postoperative recovery was uneventful, and the three-month follow-up showed no recurrence or signs of pulmonary embolism. The patient’s history of ALL raised the question of a possible association; however, a review of the literature revealed no previously reported link. In conclusion, right ventricular myxomas are extremely rare. The occurrence of cardiac myxoma in this patient following childhood ALL appears to be incidental. Further research is needed to determine whether ALL survivors have an increased predisposition to subsequent cardiac tumors.

## 1. Introduction

Primary cardiac tumors are exceedingly rare, with an estimated incidence between 0.001% and 0.3% in autopsy series [1]. The majority, approximately 75% to 90%, are benign, most commonly cardiac myxomas, which typically originate in the left atrium [2]. A recent systematic review of medical literature [3] reported a total of 174 cases of surgically treated primary left ventricular myxomas. In contrast, right ventricular myxomas are even rarer [4], with reports often limited to isolated case studies. These are significantly more uncommon than myxomas in the left ventricle. An analysis covering cases from 2001 to 2022 [5] found only 2 right ventricular myxomas among 244 total myxoma patients treated at a single center, compared to 26 right atrial myxomas and 216 left heart myxomas.

Clinical manifestations depend largely on tumor size, location, and mobility. Right ventricular tumors may present with exertional dyspnea, cough, syncope, or signs of right heart failure and can mimic other cardiopulmonary disorders such as pulmonary embolism or valvular disease. Diagnosis is often delayed or incidental [6,7].

Transthoracic and transesophageal echocardiography remain the first-line diagnostic tools, providing real-time visualization of intracardiac masses and their hemodynamic effects. Computed tomography and magnetic resonance imaging further characterize the lesions, delineating attachment, extent, and tissue properties, thereby guiding surgical management [8].

Surgical excision remains the treatment of choice for most benign and resectable primary cardiac tumors, both to obtain a definitive histopathological diagnosis and to prevent fatal complications such as obstruction or embolization. When complete resection is achieved, postoperative outcomes are generally favorable [9].

Herein, a rare case of a right ventricular myxoma is described in a young female who had acute lymphoblastic leukemia (ALL) in childhood. The present case report aims to outline the unusual attachment site and the high risk of life-threatening complications and to discuss the possible association between the myxoma and the prior ALL.

## 2. Case Report

A 34-year-old female presented to the Emergency Department of the Clinical County Emergency Hospital Bihor, Oradea, Romania, with complaints of exertional dyspnea and an irritative cough persisting for approximately three weeks.

Her past medical history included acute lymphoblastic leukemia at the age of 13, treated with chemotherapy and radiotherapy, and aseptic necrosis of the left lateral femoral condyle at the age of 16, treated by arthroscopic debridement. At the time of presentation, she was not receiving any chronic medication.

On physical examination in the emergency department, the patient was tachycardic but otherwise hemodynamically stable, with a grade 4/6 holosystolic murmur audible over the tricuspid valve area. The recorded vital parameters were as follows: blood pressure 123/72 mmHg, heart rate 100 beats/min, oxygen saturation 97% on ambient air, and respiratory rate 20/min. Aside from mild jugular venous distension, no other clinical signs of heart failure were evident.

**Figure 1:**
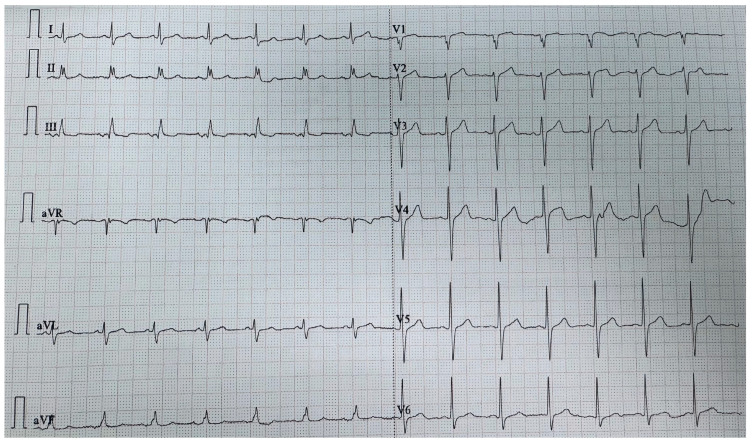
ECG at admission showing signs of right ventricular strain.

The ECG at admission, in addition to sinus tachycardia, showed a pattern consistent with acute right ventricular strain (Figure 1). Laboratory findings demonstrated a significant increase in NT-proBNP (1155 pg/mL; normal range 10.5–125.0 pg/mL) and a mild inflammatory response (C-reactive protein 6.7 mg/L; normal range 0–0.5 mg/L).

**Figure 2:**
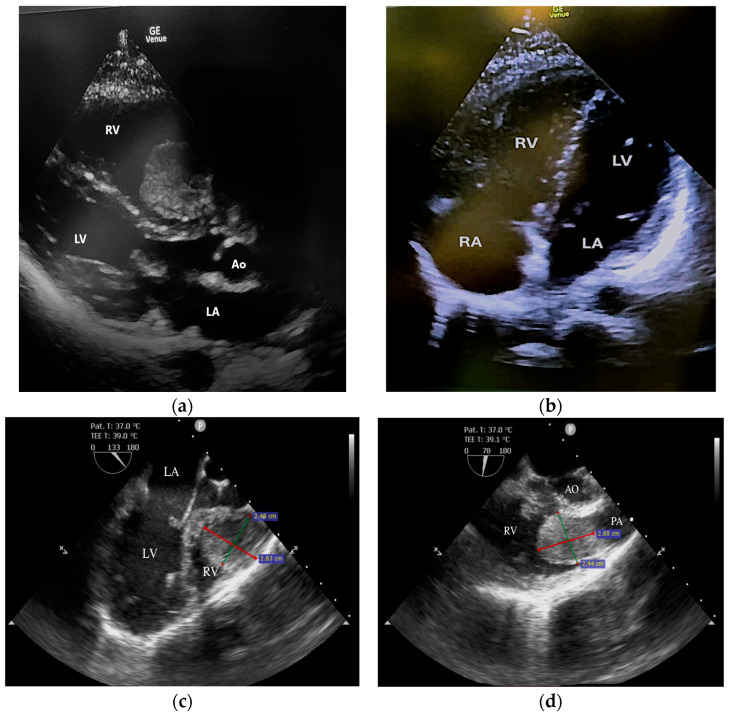
The tumoral mass identified by echocardiography: (**a**) Transthoracic echocardiography: parasternal long axis view. (**b**) Transthoracic echocardiography: apical four-chamber view. (**c**) Transesophageal echocardiography: mid-esophageal short-axis view displaying the tumor in RVOT. (**d**) Transesophageal echocardiography: mid-esophageal short-axis view displaying the tumor in the main pulmonary artery. RV, right ventricle; RA, right atrium; LV, left ventricle; LA, left atrium; PA, main pulmonary artery; RVOT, right ventricular outflow tract.

Echocardiography revealed normal left heart chambers with a preserved left ventricular ejection fraction (60%), but a dilated right atrium and ventricle, and severe tricuspid regurgitation associated with severe pulmonary hypertension. A surprising finding on echocardiography was a pedunculated tumoral mass (2.8/2.4 cm) in the right ventricle, attached to the interventricular septum, near the tricuspid valvular annulus. The tumor was visible in the parasternal long-axis view but not in the apical four-chamber view (Figure 2a,b). The transesophageal echocardiography revealed the swinging motion of the tumor back and forth through the pulmonary valve (Figure 2c,d).

**Figure 3:**
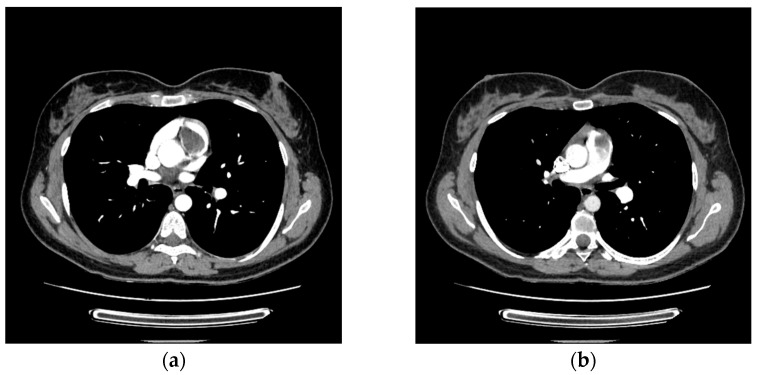
Pulmonary CT angiography displaying the tumoral mass: (**a**) in the right ventricular outflow tract; (**b**) in the main pulmonary artery.

The pulmonary CT angiography confirmed the presence of a tumoral mass in the right ventricular outflow tract, with part of it prolapsing into the main pulmonary artery. No other filling defects were detected in the pulmonary arterial tree (Figure 3a,b).

**Figure 4:**
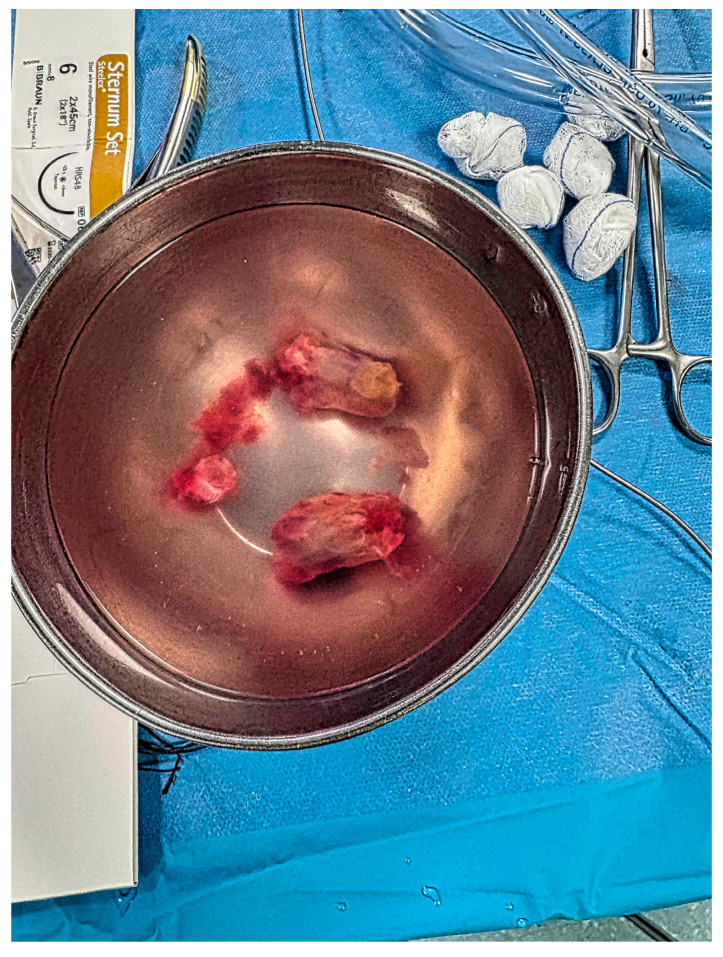
Macroscopic appearance of the excised tumoral mass.

Considering the high probability of pulmonary embolism due to possible detachment of tumoral fragments, as well as the hemodynamic impact on the right heart, the heart team decided that urgent surgery was the best therapeutic option. Within 20 h of presentation in the emergency department, the operation was performed under aorto-bicaval cardiopulmonary bypass with normothermic anterograde blood cardioplegia. During the procedure, the tumor was first pushed from the pulmonary trunk back into the right ventricle through a minimal incision in the main pulmonary artery wall. The entire mass was then removed (Figure 4) via a minimal right ventricular incision, followed by tricuspid valvuloplasty. The histopathological examination confirmed the myxomatous nature of the tumor.

**Figure 5:**
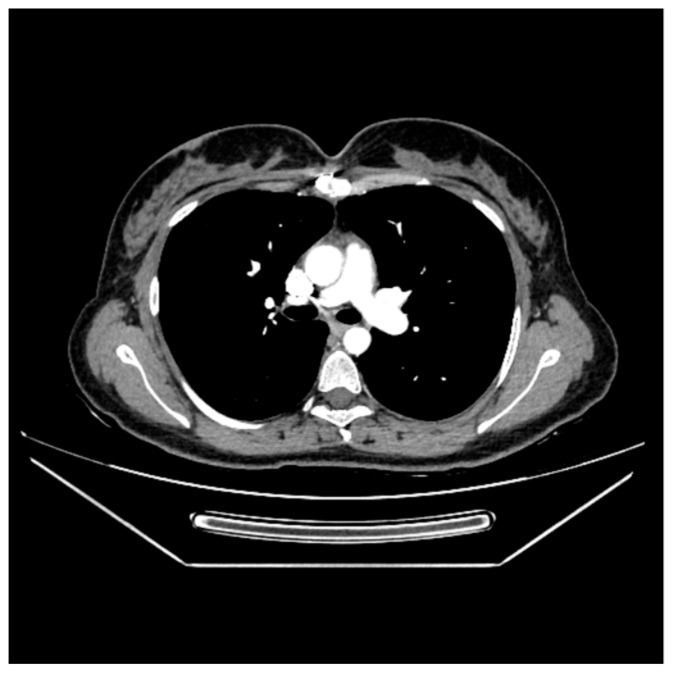
Pulmonary CT angiography at three-month follow-up, showing no pathological findings.

Postoperative recovery was uneventful, and the patient was safely discharged from the hospital on the seventh postoperative day. A pulmonary CT angiography performed three months after surgery showed no pathological findings (Figure 5).

**Figure 6:**
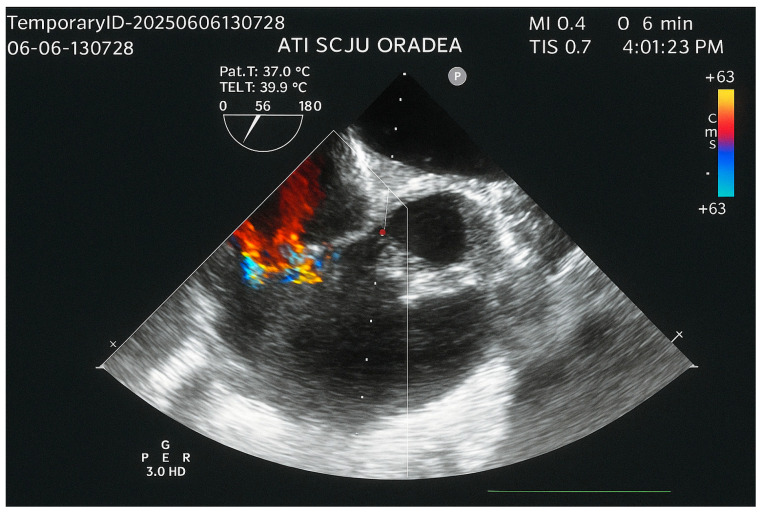
Transesophageal echocardiography, mid-esophageal short-axis view, showing moderate tricuspid regurgitation.

The transesophageal echocardiography performed one day after surgery revealed moderate tricuspid regurgitation and a significant decrease in pulmonary systolic arterial pressure (Figure 6). Follow-up echocardiograms at one and three months demonstrated persistent moderate tricuspid regurgitation and mild pulmonary hypertension.

At the three-month clinical follow-up, the patient reported no symptoms at rest and only mild exertional dyspnea. Under guideline-directed medical therapy for heart failure (including a beta-blocker, diuretic, spironolactone, SGLT2 inhibitor, and ARNI), the NT-proBNP level had decreased, nearly reaching the normal range.

**Figure 7:**
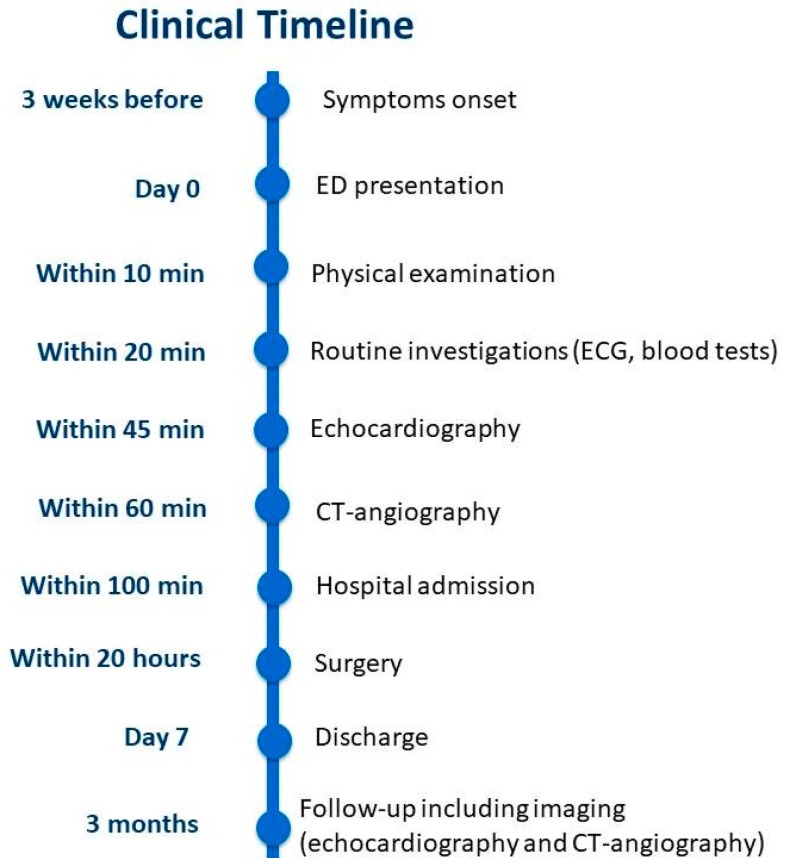
The timeline of events from symptom onset to three-month follow-up. ED, emergency department; ECG, electrocardiogram; CT, computed tomography.

A comprehensive timeline of the patient’s clinical course is provided in Figure 7.

## 3. Discussion

This case report shows a particular origin of a right ventricular myxoma occurring in a young woman with a prior hematologic malignancy. While myxomas represent most benign cardiac tumors [1], right-sided localization is uncommon [2], and right ventricular origin is particularly rare [10]. In a study conducted in Turkey between 2013 and 2024 on 274 patients with cardiac tumors, 192 (70.1%) cases proved to be cardiac myxomas [1]. Most of these originated in the left atrium and none in the right ventricle. Data in the literature on myxomas located in the RVOT are scarce. The majority of the available information is represented by case reports. According to Katiyar et al., only 30 cases have been reported over 10 years [11]. In this regard, our case report contributes new clinical data to this topic.

The delayed clinical presentation in this case aligns closely with findings reported in the literature. Cardiac myxomas, especially those located on the right side, often remain clinically silent for long periods, with symptoms appearing only when the tumor causes hemodynamic obstruction, valvular interference, or embolization [12,13].

Because myxomas typically grow slowly and their effects depend more on location and mobility than on size, many patients remain asymptomatic for months or even years [14]. In a large review by Pinede et al., approximately 20–30% of patients had symptoms for less than one month before diagnosis, whereas others experienced prolonged, vague complaints often attributed to other conditions [15].

In right-sided tumors, the situation is even more deceptive. Right atrial or ventricular myxomas can mimic pulmonary or respiratory diseases, presenting with dyspnea, cough, or fatigue due to intermittent right ventricular outflow obstruction or pulmonary embolization [16]. Several reports have noted that symptoms often appear abruptly, typically when the tumor becomes mobile enough to prolapse through the tricuspid valve or right ventricular outflow tract, as occurred in our patient [17,18]. Therefore, the three-week symptomatic interval described here is entirely consistent with published data, representing the late, mechanical phase of tumor evolution, when dynamic obstruction or embolic phenomena begin to manifest clinically.

Complete excision is crucial to prevent recurrence, but the tumor’s pedunculated attachment near the septal endocardium necessitated meticulous resection to preserve myocardial integrity and avoid injury to the conduction system [15]. The employed surgical strategy (the initial repositioning into the right ventricle through a minimal pulmonary artery incision, followed by complete excision via a right ventricular approach) intended to minimize the risk of tumor fragmentation and pulmonary embolization during manipulation, as described in the literature [16]. The concomitant tricuspid valvuloplasty addressed the regurgitation induced by annular dilation and leaflet traction from the tumor’s motion. Postoperative echocardiography confirmed significant improvement in right ventricular pressures and only moderate residual tricuspid regurgitation, reflecting successful hemodynamic restoration. Long-term prognosis after complete surgical excision of right-sided myxomas is excellent, though recurrence (usually due to incomplete resection) can occur in a small proportion of cases [17]. Serial echocardiographic follow-up remains essential to detect recurrence early, the association of the echocardiographic parameters being strongly related to the patient’s quality of life [18,19]. Up to the moment the case report was written (four months of follow-up), the serial echocardiograms showed no signs of recurrence. As the patient presented with right heart failure, her in-hospital and discharge medication regimen included the four pillars of guideline-directed medical therapy for heart failure [20]. At the three-month clinical follow-up, as she showed clinical and paraclinical signs of improvement, a progressive tapering of this therapy was considered.

Our patient had a history of acute lymphoblastic leukemia treated with chemotherapy and radiotherapy. Aseptic necrosis of the femoral condyle she later developed is a known late effect of cancer-related therapy [21]. In this regard, we questioned whether the current myxoma could be related to her prior ALL.

Cardiac complications related to chemotherapy typically include congestive heart failure, coronary artery disease, myocardial infarction, cardiac arrest, and cerebrovascular accidents [20,22]. The cardiotoxic effects of radiotherapy are mainly the result of vascular damage and fibrosis, mediated by various acute and late-acting inflammatory cytokines. They are represented by pericarditis, conduction disorders, coronary artery disease, valvular heart disease, and cardiomyopathy [23].

According to Wang et al., childhood cancer survivors have an increased risk of developing second malignant or benign neoplasms later in life [24]. The incidence of a second neoplasm in childhood ALL survivors is low, approximately 2–4% [25]. The types of the second tumors described comprise central nervous system neoplasms, fibroblastic sarcoma, lymphoma, thyroid carcinoma, basal cell carcinoma, adenocarcinoma, and squamous cell carcinoma [25,26]. To our knowledge, there are no published studies or reviews demonstrating an increased risk of myxomas following acute lymphoblastic leukemia [27]. While hematologic malignancies may secondarily involve the heart through metastasis or infiltrative disease [28,29] and isolated reports exist describing secondary cardiac tumors in patients with hematologic disorders [30], none suggests an association between ALL and cardiac myxomas. Moreover, the long time elapsed between the ALL episode and the present condition (more than 20 years) makes the association less likely.

Given the rarity of cardiac myxomas, the well-documented late cardiotoxic effects of ALL therapy, and the lack of evidence linking ALL to cardiac myxomas, we concluded that the coexistence observed in our patient was most probably coincidental. Nevertheless, this case highlights the importance of long-term follow-up in survivors of childhood malignancy [21] to allow early detection of late therapy-related complications, including secondary tumors, and to prevent potentially life-threatening consequences.

ALL was previously regarded as a non-hereditary disease. However, recent genomic studies in both familial and sporadic cases have identified genetic variants associated with ALL development and with therapy-related secondary malignancies [31]. Further research is needed to clarify the potential genetic predisposition linking ALL and subsequent tumors. In the future, such studies may identify that an association between ALL and cardiac myxomas, as secondary tumors, truly exists.

## 4. Conclusions

Right ventricular cardiac myxomas are exceedingly rare. The present case report contributes to the limited clinical literature about right ventricular myxomas by documenting a rare site of attachment. In our patient, the occurrence of a cardiac myxoma following a previous diagnosis of ALL appeared to be incidental. Nevertheless, further genetic investigations may help clarify the mechanisms underlying secondary malignancies in survivors of childhood ALL, particularly in light of emerging evidence suggesting that genetic predisposition plays a role in their increased long-term oncologic risk.

## Data Availability

The original contributions presented in this study are included in the article. Further inquiries can be directed to the corresponding author.

## Abbreviations

The following abbreviations are used in this manuscript:

ECG	Electrocardiogram
NT-proBNP	N-terminal pro-B-type natriuretic peptide
RV	Right ventricle
RA	Right atrium
LV	Left ventricle
LA	Left atrium
PA	Main pulmonary artery
RVOT	Right ventricular outflow tract
CT	Computed tomography
SGLT2	Sodium-Glucose Cotransporter-2
ARNI	Angiotensin receptor-neprilysin inhibitor
ALL	Acute lymphoblastic leukemia
